# Social Media Use Among Orthopedic and Trauma Surgeons in Germany: Cross-Sectional Survey Study

**DOI:** 10.2196/45665

**Published:** 2023-09-22

**Authors:** Yasmin Youssef, Julian Scherer, Marcel Niemann, Jörg Ansorg, David Alexander Back, Tobias Gehlen

**Affiliations:** 1 Department of Orthopaedics, Trauma and Reconstructive Surgery University Hospital Leipzig Leipzig Germany; 2 Department for Traumatology and Orthopedics Bundeswehr Hospital Berlin Berlin Germany; 3 Department of Traumatology University Hospital of Zurich Zurich Switzerland; 4 Orthopaedic Research Unit University of Cape Town Cape Town South Africa; 5 Center for Musculoskeletal Surgery Charité - Universitätsmedizin Berlin Berlin Germany; 6 Akademie Deutscher Orthopäden (German Academy of Orthopedists) Berufsverband für Orthopädie und Unfallchirurgie e. V. (Professional Association of Specialists in Orthopedic and Trauma Surgery) Berlin Germany; 7 Dieter Scheffner Center for Medical Education and Educational Research Charité - Universitätsmedizin Berlin Berlin Germany

**Keywords:** communication, digitalization, Facebook, Germany, implementation, Instagram, management, musculoskeletal, orthopedic surgeon, orthopedic, orthopedics and traumatology, patient, physician, questionnaire, social media management, social media, social networking, surgeon, trauma surgeon

## Abstract

**Background:**

Social media (SM) has gained importance in the health care sector as a means of communication and a source of information for physicians and patients. However, the scope of professional SM use by orthopedic and trauma surgeons remains largely unknown.

**Objective:**

This study presents an overview of professional SM use among orthopedic and trauma surgeons in Germany in terms of the platforms used, frequency of use, and SM content management.

**Methods:**

We developed a web-based questionnaire with 33 variables and 2 separate sections based on a review of current literature. This study analyzed the first section of the questionnaire and included questions on demographics, type of SM used, frequency of use, and SM content management. Statistical analysis was performed using SPSS (version 26.0). Subgroup analysis was performed for sex, age groups (<60 years vs ≥60 years), and type of workplace (practice vs hospital). Differences between groups were assessed with a chi-square test for categorical data.

**Results:**

A total of 208 participants answered the questionnaire (166/208, 79.8% male), of whom 70.2% (146/208) were younger than 60 years and 77.4% (161/208) worked in a practice. All participants stated that they use SM for private and professional purposes. On average, participants used 1.6 SM platforms for professional purposes. More than half had separate SM accounts for private and professional use. The most frequently used SM platforms were messenger apps (119/200, 59.5%), employment-oriented SM (60/200, 30%), and YouTube (54/200, 27%). All other SM, including Facebook and Instagram, were only used by a minority of the participants. Women and younger participants were more likely to use Instagram (*P*<.001 and *P*=.03, respectively). The participants working in a hospital were more likely to use employment-oriented SM (*P*=.02) and messenger apps (*P*=.009) than participants working in a practice. In a professional context, 20.2% (39/193) of the participants produced their own content on SM, 24.9% (48/193) used SM daily, 39.9% (77/193) used SM during work, and 13.8% (26/188) stated that they checked the number of followers they had. Younger participants were more likely to have participated in professional SM training and to have separate private and professional accounts (*P*=.04 and *P*=.02, respectively). Younger participants tended toward increased production of their own content (*P*=.06).

**Conclusions:**

SM is commonly used for professional purposes by orthopedic and trauma surgeons in Germany. However, it seems that professional SM use is not exploited to its full potential, and a structured implementation into daily professional work routines is still lacking. SM can have a profound impact on medical practices and communication, so orthopedic and trauma surgeons in Germany should consider increasing their SM presence by actively contributing to SM.

## Introduction

The popularity of social media (SM) continuously grows, and it has started to affect almost all areas of our daily lives [1-3]. The term SM refers to all digital applications that allow people to communicate, produce, share, or comment on content using the internet or mobile apps [4]. The advantages of SM are the low barrier to interactions between users and the distribution of information from almost anywhere at any time. In 2022, the most popular SM platforms worldwide were Facebook, YouTube, WhatsApp, and Instagram [5].

Recently, SM has also gained importance in the health care sector. According to some studies, up to 88% of health care providers use SM on a regular basis [6-9]. The use of SM in the health care sector by individual physicians and institutional providers like hospitals, societies, and scientific journals can help to facilitate and improve peer-to-peer and clinician-to-patient communication, amplify information outreach, educate patients, promote institutional branding, and increase publicity [6-12]. Furthermore, a growing number of patients use SM when seeking information on health care providers, health conditions, and treatment options [6,13-15]. However, there are also potential dangers of SM use in the medical sector, including violation of patient privacy, as well as concerns about medicolegal, confidentiality, and liability aspects [8-10,16]. Furthermore, SM also incorporates the risk of spreading health-related misinformation, as could be seen during the COVID-19 pandemic [17-19].

Recent studies have also shown that orthopedic patients increasingly use internet applications and SM [20,21]. This underlines the sense of why orthopedic and trauma surgeons should deal with this new form of interaction with their patients. Duymus et al [21] found that 48.5% of patients preferred mobile apps that allow direct interaction with a physician over apps that do not allow interaction. Further, 34.2% had already contacted an orthopedic or trauma surgeon over the internet [21]. Internet research influenced both hospital and physician choices of patients in up to 50.9% and 39.4% of cases, respectively [21]. Previous data on the user behavior of orthopedic and trauma surgeons showed that LinkedIn and Facebook are the most frequently used platforms [22-27], while Instagram was shown to be used more frequently by younger professionals [7]. There is no sufficient data on the use of Snapchat and TikTok. Even though the professional use of SM in the field of orthopedic and trauma surgery seems promising, a collective and thorough understanding of how SM is and could be used in orthopedic and trauma surgery is lacking. Detailed data on consumption and use behavior related to the professional use of SM in the field of orthopedic and trauma surgery are needed [28,29]. Thus, the aim of this study was to present an overview of the use of SM among German orthopedic and trauma surgeons regarding the types of platforms used as well as the usage behavior for professional purposes.

## Methods

### Study Design

A web-based questionnaire was created to assess the current use of SM among orthopedic and trauma surgeons in Germany and administered using SurveyMonkey (SurveyMonkey). The questionnaire was sent to members of the BVOU (Berufsverband für Orthopädie und Unfallchirurgie; Professional Association of Orthopedic Surgeons in Germany) through their email distributor. The survey was conducted between June 2022 and July 2022.

### Questionnaire

The questionnaire was developed by the study team based on a review of current literature [22-27,30] and was complemented with further aspects of interest. The preliminary questionnaire was pretested among 5 independent orthopedic and trauma surgeons. The questionnaire was finalized considering the feedback from this pilot group. The final questionnaire (Multimedia Appendix 1) consisted of 33 variables and included 2 separate parts. In the first section, which concentrated on the types of SM platforms used for private and professional purposes, use behavior and content management were addressed. The second section included the purposes of SM use as well as the perceived benefits and difficulties. This presented study only analyzed the data from the first section of the questionnaire, as full coverage would have exceeded the limits of this study.

### Data Processing and Statistical Analysis

Statistical analysis was performed using SPSS (version 26.0; IBM Corp). Categorial data are presented as frequencies and percentages. Subgroup analyses were performed for sex (male vs female), age (<60 years vs ≥60 years), and type of workplace (practice vs hospital). To assess differences between groups, the chi-square test was used for categorical data. The level of statistical significance was set at a 2-sided *P* value of <.05.

### Ethical Considerations

All participants received written patient information that explained the aim and scope of the study as well as how data would be collected, processed, and analyzed. Participation was voluntary, and anonymity was granted as no identifying data except for age, gender, and occupation were collected. Hence, it can be assumed to be anonymous, and the European data protection regulations [31] do not apply. Participants gave their consent to process the data provided by answering the questionnaire. No formal ethical approval by an ethics committee was needed for the conduct of this study, as general waivers apply for surveys with anonymous data in Germany.

## Results

### Demographics

In total, 208 participants answered the questionnaire (166/208, 79.8% male), of whom 70.2% (146/208) were aged <60 years. Most women were aged <60 years (39/42, 92% <60 years old vs 3/42, 4% ≥60 years old; *P*<.001). Younger participants were more likely to work in hospitals (39/47, 83% <60 years old vs 8/47, 17% ≥60 years old; *P*=.03). More than half of the participants (161/208, 77.4%) worked in a practice, and 22.6% (47/208) worked at a hospital. Men were more likely to work in a practice (138/161, 85.7% men vs 23/161, 14.3% women; *P*<.001; Table 1).

**Table 1:** Demographics stratified by age group, gender, and type of workplace (N=208).

	Practice			Hospital	
	Men (n=138), n (%)	Women (n=23), n (%)	Men (n=28), n (%)		Women (n=19), n (%)
<60 years old	86 (41.8)	21 (10)	21 (10)		18 (8)
≥60 years old	52 (25)	2 (1)	7 (3)		1 (0.5)

### Number of SM Platforms Used

A total of 6 participants did not answer the questions on private SM use. The questions about using SM professionally were not answered by 8 participants. On average, participants used 2.6 (range 0-9) SM platforms for private purposes and 1.6 (range 0-6) for professional purposes.

The lowest number of SM platforms used for professional purposes was observed in participants aged ≥60 years (mean 1.3; range 0-4), while the highest number was among women and participants working at a hospital (mean 2.3; range 0-6, respectively).

### SM Platforms Used for Private Purposes

All participants (202/202) used SM for private purposes. The SM platforms most frequently used were messenger apps (174/202, 86.1%), YouTube (115/202, 56.9%), employment-oriented SM (eg, Xing and LinkedIn; 72/202, 35.6%), and Facebook (66/202, 32.7%).

Women were more likely to use Facebook (46/164, 28% men vs 20/38, 52% women; *P*=.004) and Instagram (30/164, 18.3% men vs 19/38, 50% women; *P*<.001). Participants working at a hospital were more likely to use Facebook (21/43, 48% hospital vs 45/159, 28.3% practice; *P*=.01), Instagram (19/43, 39% hospital vs 39/159, 20.1% practice; *P*=.008), and employment-oriented SM (21/43, 48% hospital vs 51/159, 32.1% practice; *P*=.04). Younger participants were more likely to use Facebook (56/141, 39.7% <60 years old vs 10/61, 16% ≥60 years old; *P*=.001), Instagram (42/141, 29.8% <60 years old vs 7/61, 11% ≥60 years old; *P*=.005), and messenger apps (128/141, 90.8% <60 years old vs 46/61, 76% ≥60 years old; *P*=.007).

### SM Platforms Used for Professional Purposes

All participants (200/200) used SM for professional purposes. The SM platforms most frequently used were messenger apps (119/200, 59.5%), employment-oriented SM (60/200, 30%), and YouTube (54/200, 27%). Further, most participants used websites (138/200, 71.1%) for professional purposes. Podcasts and blogs were used by 25.3% (49/194) and 8.2% (16/194) of the participants, respectively.

The use of Instagram was higher among women (10/164, 6.1% men vs 11/36, 30% women; *P*<.001) and younger participants (19/139, 13.7% <60 years old vs 2/61, 3% >60 years old; *P*=.03). Participants working at a hospital were more likely to use employment-oriented SM (19/43, 44% hospital vs 41/157, 26.1% practice; *P*=.02) and messenger apps (33/43, 76% hospital vs 86/157, 54.8% practice; *P*=.009). Overall, 41% (16/39) of the participants working at hospitals used podcasts, compared to 21.3% (33/155) of the participants working in a practice (*P*=.01).

### Frequency of SM Use for Professional Purposes

For professional purposes, 24.9% (48/193) of the participants used SM daily, 10.9% (21/193) used it infrequently, and 16.6% (32/193) never used it. The highest frequency of use was observed among women (13/35, 37%). There were no differences detected in the usage frequency for the subgroups.

More than a third of the participants (77/193, 39.9%) used SM at work. Participants working at hospitals (20/37, 54% hospital vs 57/156, 36.5% practice; *P*=.05) and young participants (60/135, 44.4% <60 years old vs 17/58, 29% ≥60 years old; *P*=.049) were more likely to use SM at work. There was no significant difference between men and women (*P*=.99).

### Content Management of SM

Most participants (107/190, 56.3%) had separate SM accounts for private and professional use. Younger participants were more likely to have separate SM accounts (82/133, 61.7% <60 years old vs 25/57, 43% ≥60 years old; *P*=.02).

Only 20.2% (39/193) created their own content on SM, with a tendency toward higher content production among younger participants, although this was not significant (32/134, 23.9% <60 years old vs 7/59, 11% ≥60 years old; *P*=.06). Overall, 13.8% (26/188) regularly checked their number of followers, and 7.8% (15/192) had already taken part in training concerning professional use of SM. Younger participants were more likely to have taken part in such training (14/134, 10.4% <60 years old vs 1/59, 1% ≥60 years old; *P*=.04).

## Discussion

### Overview

The aim of this study was to systematically assess the user behavior of German orthopedic and trauma surgeons regarding the implementation of SM in their private and professional lives. In addition, the influence of age, gender, and workplace environment on user behavior was analyzed.

First, the results indicate that the use of SM is common among orthopedic and trauma surgeons in Germany. All participants stated that they use SM both for private and professional purposes. The presented use rates for professional purposes are much higher compared to previous studies in the fields of orthopedics and trauma surgery. Previous studies found rates of SM use between 37% and 65.7% [7,22,23,25,26,32,33]. The heterogeneity in previous studies could be explained by regional differences in SM use as well as the time of study conduct, which included studies between 2013 and 2021. The latter is of utmost importance, as digitalization and the availability of web-based services continuously progresses, and accordingly, it is expected that their use naturally increases over time. However, it must also be noted that there is a certain incongruence in our findings, as while all participants stated that they use SM for professional purposes, 16.6% (32/193) stated that they never use it in the question on the frequency of use.

Second, 56.3% (107/190) of our participants stated that they have separate private and professional accounts. Fewer SM accounts were used for professional than private purposes. To the best of the authors’ knowledge, no previous study assessed differences in platform use for private and professional purposes and whether separate accounts are used by orthopedic and trauma surgeons. Our findings on the SM platforms used are comparable to those presented in previous studies, even if these did not include messenger apps and only a minority analyzed YouTube and TikTok use. The platforms most frequently used for professional purposes were messenger apps, employment-oriented SM, and YouTube. All other media, including Facebook and Instagram, were only used by a minority of the participants for professional purposes. On the other hand, websites were still used by most of the participants. Garofolo et al [22,23] also reported high website use.

Previous studies also showed that the use of employment-oriented SM like LinkedIn, Facebook, and YouTube is high among orthopedic and trauma surgeons [22-26]. On the other hand, only 3% (6/200) of our participants stated that they use Twitter for professional purposes, whereas previous studies reported rates between 12.4% and 75.9% [7,23,24,26]. This could be explained by the lacking differentiation between private and professional use in other studies, as well as the country of study conduct, which was the United States in most cases. The United States has the highest number of active Twitter users, while its use is not as widespread in Germany [34]. However, it must be noted that in the study cohort that reported a 75.9% use of Twitter, 90% of the participants were aged <40 years [7].

In accordance with previous data, young participants were more likely to use Instagram [7]. This was not the case for Facebook, Twitter, employment-oriented SM, and YouTube. This corresponds to a report by Garofolo et al [22,23]. Further, differences in use behavior between participants working in a practice and in a hospital were observed. In part, this could be explained by the age differences we observed between these groups. On the other hand, it could reflect the differences in the SM requirements at the 2 workplaces. It could be that participants working in a hospital use more messenger apps and employment-oriented SM, as they have a greater need for networking for reasons such as research. Further studies should be conducted to determine the motivation and perceived advantages of the different SM platforms used.

Third, whereas all participants stated that they use SM, the potential of their professional use does not seem to be exploited to its fullest. In total, only 24.9% (48/193) of the participants used SM daily, and 10.9% (21/193) used it infrequently for professional purposes. Justinia et al [7], on the other hand, found that 74.7% of their study cohort used SM daily. However, it must be noted that these participants were younger compared to our cohort. Furthermore, only a minority of the participants (32/193, 20.2%) stated that they produce their own content on SM, and only 13.8% (26/188) checked the number of followers that they have. These results show that SM use among orthopedic and trauma surgeons in Germany is mainly passive. This might be explained by the legal force of the European General Data Protection Regulation (GDPR) in Germany and unclear regulations on professional SM use (eg, case presentation on SM) by physicians. The GDPR regulates the use of personal data of all European Union citizens and applies to all organizations and companies that process personal data, including the medical sector [35]. According to these regulations, patients must clearly consent before any patient data (eg, medical history, lab values, and images) can be analyzed, saved, or shared. In addition to that, patient data must be saved and exchanged safely, inhibiting any form of data leakage or misuse [35]. SM platforms such as Zoom, Skype, and WhatsApp do not fulfill the digital safety requirements and their use is prosecutable. In addition to that, physicians must guarantee that all data can be deleted if demanded by the patient. This might pose a reason for insecurity in the use of professional SM for many physicians. The accepted scope of interaction, suitable SM platforms, and the information or data that are allowed to be shared through SM in a professional context might not be fully clear for all users. Future studies will be needed to identify the perceived medicolegal risks of professional SM use.

Producing one’s own content on SM can affect one’s visibility on SM and, therefore, in the community. Zhang and Earp [12] investigated SM posts and academic citations of recent orthopedic research. Similarly, Kunze et al [11] found that attention to content on SM was associated with higher citation numbers. In addition, patients increasingly use SM. Among orthopedic patients, Duymus et al [21] were able to show that a participant’s choice of a hospital or orthopedic surgeon was affected by their use of the internet in 50.9% and 39.4% of cases, respectively. Curry et al [20] found that 50% of their patient cohort used SM, including Facebook and Twitter, with the highest proportion of SM users being sports medicine patients.

Considering the presented trends, the authors suggest that SM use among orthopedic and trauma surgeons in Germany should be further developed, as it could help to promote individual and public health, improve patient-clinician communication, and promote professional development and advancement [6,8-12]. Further research on consumption and use behavior will be needed. In addition to that, further research on the reasons for use, including motivation for its implementation and perceived possibilities and difficulties, should be conducted.

This study has certain limitations. First, it must be noted that surveys have minor levels of evidence and that the outcome can be affected considerably by the participants’ understanding of the questionnaire. Therefore, trends in the presented results must be treated with caution, also considering the unequal sizes of the subgroups and broad age spectrum. Furthermore, it must be noted that we did not differentiate between orthopedic and trauma surgeons. This can be justified since orthopedics and trauma surgery are a joint specialty in Germany. In addition, due to the voluntary nature of their participation, orthopedic and trauma surgeons with a critical attitude toward SM use for professional purposes might be underrepresented, which might result in bias.

### Conclusion

SM is commonly used by orthopedic and trauma surgeons for professional purposes in Germany. However, it seems that professional SM use is not exploited to its full potential, and a structured implementation into daily professional work routines is still lacking. SM can have a profound impact on medical practices and communication; orthopedic and trauma surgeons in Germany should thus consider increasing their SM presence by actively contributing on SM.

## Appendix

Multimedia Appendix 1 Questionnaire - English version.

## Abbreviations

GDPR: General Data Protection Regulation
SM: social media

## Data Availability

The data sets generated during and/or analyzed during this study are available from the corresponding author on reasonable request.
