# Supplementary material for: Social Media Use Among Orthopedic and Trauma Surgeons in Germany: Cross-Sectional Survey Study
Source: JMIR Form Res. 2023 Sep 22;7:e45665. doi: 10.2196/45665 (PMC10559187; doi:10.2196/45665)
Supplement: Multimedia Appendix 1 [file formative_v7i1e45665_app1.pdf]

Questionnaire on the use of Social Media among orthopaedic and trauma surgeons in Germany

Sociodemographic Data

1. Gender:
   - Male
   - Female
   - Diverse

2. Age:
   - < 30
   - 31-40
   - 41-50
   - 51-60
   - > 60

3. Localization of your workplace:
   - Large city (> 100,000 inhabitants)
   - Middle sized town (population between 20,000 and 100,000 inhabitants)
   - Small town (Population < 20,000 inhabitants)
   - Rural community

4. Level of professional training:
   - Resident
   - junior consultant
   - consultant
   - senior consultant
   - consultant in practice

5. In what type of your workplace do you work?
   - medical center
   - Practice
   - hospital

6. How many employees work in your workplace/department?
   - 1-4
   - 5-10
   - >10
   - >20
   - >30
   - >40

Type of Media used

7. Which social media do you use for private purposes? (multiple answers possible)
   - Twitter
   - Facebook
   - Instagram
   - TikTok
   - employment oriented social networks such as LinkedIn or Xing
   - Messenger Apps (WhatsApp, Telegram, Signal etc.)
   - YouTube
   - Other (please specify)
   - None of the above

8. Which social media do you personally use in your professional environment? (multiple answers possible)
   - Twitter
   - Facebook
   - Instagram
   - TikTok
   - employment oriented social networks such as LinkedIn or Xing
   - Messenger Apps (WhatsApp, Telegram, Signal etc.)
   - YouTube
   - Other (please specify)
   - None of the above

9. What other digital media do you use professionally? (multiple answers possible)
   - podcast
   - blog
   - website
   - Waiting room TV
   - Other (please specify)
   - None of the above

Management of professional social media accounts and usage behavior

10. Who is in your practice/department is responsible for updating content on your professional social media accounts (e.g. new posts or replying to patient inquiries) (multiple answers possible)
    - Myself
    - Other internal medical staff
    - Other internal non-medical personnel
    - External Vendors
    - Other (please specify)
    - None of the above

11. Do you have separate social media accounts for professional and private purposes?
   - Yes
   - No

12. Have you ever attended a course on the topic of social media for Professionals purposes?
   - Yes
   - No

13. How frequently do you use social media for professional purposes?
   - Daily
   - Several times a week
   - Once a week
   - Weekends only
   - At least once a month
   - Infrequently
   - Never

14. I produce own content on diseases/treatment methods.
   - Yes
   - No

15. Do you use your professional social media accounts during work hours?
   - Yes
   - No

16. Do you check how many followers you have on your professional social media account?
   - Yes
   - No

17. If so, how do you react to changing follower numbers?
   - When the number of followers is low, I increase my post frequency
   - If I have a high number of followers, I will keep my post frequency
   - I do not match my post frequency with my follower count
   - Other (please specify)
   - None of the above

Professional uses of social media

18. For what professional purposes do you use the following social media channels?

	Facebook	Twitter	Instagram	TikTok	Employment oriented social media	Messenger Apps	YouTube	Website

For conducting further training (also CME...).								
For receiving health-related information.								
For professional networking (networking with colleagues).								
For sharing health-related information.								
For sharing own clinical experience and expertise.								
To produce and post your own content about diseases and treatment methods.								
To communicate with patients.								
To acquire new patients or to keep existing ones.								

Perceived advantages of social media uses

19. What advantages do you see in the use of social media in a professional context?

	Strongly agree	agree	Neutral	Disagree	Strongly disagree
Social media help me acquire patients for my practice/department.					
Social media help me communicate and interact with my patients (e.g. for receiving and responding to patient inquiries, criticism and opinions).					
Social media help me to keep up to date with the latest developments in my field.					
Social media help me to present the offers of my practice/department.					

Perceived difficulties of social media use

20. What difficulties do you see in the use of social media in a professional context?

	Strongly agree	agree	Neutral	Disagree	Strongly disagree
Using social media for professional purposes is time-consuming.					

I have insufficient knowledge on how to use social media efficiently for professional purposes.					
I feel unsure about legal regulations regarding data protection when using social media for professional purposes.					
I find it difficult to assess which content on social media appeals to patients.					
